# National health insurance, social influence and antenatal care use in Ghana

**DOI:** 10.1186/2191-1991-3-19

**Published:** 2013-08-06

**Authors:** Nkechi S Owoo, Monica P Lambon-Quayefio

**Affiliations:** 1 Department of Economics, University of Ghana, P. O. Box LG 57, Legon, Accra, Ghana; 2 Department of Economics, Clark University, 950 Main Street, Worcester, MA, 01610, USA

**Keywords:** Antenatal care, Health insurance, Social influence, Child and maternal health, Maternal mortality, Spatial analysis

## Abstract

The study explores the importance of social influence and the availability of health insurance on maternal care utilization in Ghana through the use of antenatal care services. A number of studies have found that access to health insurance plays a critical role in women’s decision to utilize antenatal care services. However, little is known about the role that social forces play in this decision. This study uses village-level data from the 2008 Ghana Demographic and Health Survey to investigate the effects of health insurance and social influences on the intensity of antenatal care utilization by Ghanaian women. Using GIS information at the village level, we employ a spatial lag regression model in this study. Results indicate that, controlling for a host of socioeconomic and geographical factors, women who have health insurance appear to use more antenatal services than women who do not. In addition, the intensity of antenatal visits appears to be spatially correlated among the survey villages, implying that there may be some social influences that affect a woman’s decision to utilize antenatal care. A reason for this may be that women who benefit from antenatal care through positive pregnancy outcomes may pass this information along to their peers who also increase their use of these services in response. Traditional/Cultural leaders as “gate-keepers” may be useful in the dissemination of maternal health care information. Public health officials may also explore the possibility of disseminating information relating to maternal care services via the mass media.

## Background

The importance of maternal health services in reducing maternal and infant mortality has been emphasized in a number of studies [1-5]. Indeed, under-utilization of health services has been mentioned as the major factor in maternal mortality in developing countries [2]. In developing countries maternal mortality rates are estimated at about 480 deaths per 100,000 live births, and over half a million women die each year from complications relating to pregnancy and childbirth [6].

The importance of maternal health is emphasized in the Fifth Millennium Development goal which aims to reduce maternal deaths by three quarters by 2015- the current maternal mortality ratio in Ghana is 350 per 100,000 live births, according to the CIA World Factbook. The 2008 Ghana Demographic and Health Survey (GDHS) report [7] emphasizes that the health care that a mother receives during and after delivery is a crucial factor in the survival of both mother and child. This is because during an antenatal visit, pregnant women are screened and treated for possible complications during pregnancy. In addition, pregnant women are given vital information on diet and other general safety practices for mother and child.

The World Health Organization recommends that women attend a minimum of four antenatal visits, with the first visit made in the first trimester of pregnancy. Obstetricians generally recommend monthly antenatal visits up to the seventh month of pregnancy, bi-weekly visits up to the eighth month, and weekly visits thereafter. Assuming women make their first antenatal visit in the third month of pregnancy, this translates to about 12-13 antenatal visits for the duration of a pregnancy [7]. In Ghana, over 90% of women report seeing a health professional at least once during pregnancy for the most recent birth in the five-year period before the survey. This figure varies by the age of the mother, education status, rural/urban residence and region of residence, with coverage declining among older women, less educated women and women who reside in rural areas.

A barrier to the use of maternal health services in Ghana is the lack of affordability and accessibility of the health care system [8]. According to Overbosch et al. [9] and Abor and Abekah-Nkrumah [10], wealth has a positive and significant impact on antenatal care use in Ghana. This is to be expected as antenatal care involves the cost of consultation, purchase of recommended drugs, and treatment of possible pregnancy complications, among others. Several other studies have emphasized the importance of wealth on maternal health service utilization. For instance, in Ghana, Abor and Abekah-Nkrumah [10] find that richer women are more likely to use antenatal services. Arthur [5] also finds that wealth has a significant influence on the adequate use of antenatal care, using data from the 2008 ‘Ghana Health and Demographic Survey.

In order to solve the problems of inaccessibility and unaffordability, the free maternal health policy was initiated in September 2003 in four (4) regions of Ghana- Central, Upper East, Upper West, Northern- and later extended to the remaining six (6) regions in April, 2005. Women were required to register with the National Health Insurance scheme in order to obtain access to the free maternal health care. This policy was intended to reduce the financial costs involved in obtaining health care during and after pregnancy.

Few studies have examined the impact of free maternal health policy on antenatal care utilization. However, these studies have consistently found a positive and significant relationship between health insurance and antenatal care utilization. Chen et al. [11] compare access to antenatal care services before and after the implementation of the national health insurance scheme in Taiwan and find that after the policy, the number of antenatal care visits increased. Mensah et al. [12] find that women enrolled in the national health insurance scheme in Ghana are more likely to seek maternal health care. Brugiavini and Pace [13] also find that the introduction of the National Health Insurance Scheme has a positive and significant effect on the utilization of health care services in Ghana.

No study that we are aware of has attempted to empirically examine the role played by social forces in influencing women’s decision to utilise antenatal care services in Ghana. The communal nature of living in the Ghanaian society pre-disposes individuals to the continuous exchange of information and ideas, and the potential for social influence. With respect to the decision of whether or not to utilize antenatal care services, women may have obtained information about favourable pregnancy outcomes through early detection of possible complication from their social networks, and therefore may be influenced to also patronize these maternal health services themselves. At the very least, other women may not wish to be left out and therefore patronize these antenatal care services in the interest of conformity.

This study seeks to contribute to the existing literature on maternal health services utilization and fill the research gap by investigating the potential role that social interactions play in influencing women’s decision to patronize these services. The study uses GIS and village-level data from the Ghana Health and Demographic Survey (2008) and employs simple OLS and spatial lag estimation models in the analysis. A total of 394 villages are included in this study.

The paper is structured as follows. We provide a brief historical context of health insurance in Ghana, and the potential contribution of social factors to maternal care use. Descriptive data is presented at the village level on the set of socioeconomic and geographic variables used in the empirical analyses. This is followed by a description of the empirical specification, including some exploratory spatial data analysis. Estimates of the effect of health insurance and social influence on antenatal care utilization under both OLS and spatial lag empirical specifications are discussed after which conclusions and policy suggestions will be presented.

### Historical context of health insurance

During the colonial era, Ghana’s health care operated with direct payments at points of service, which were usually hospitals. This benefitted the elite colonials, while the rest of the population relied on traditional and missionary sources of health care. After independence, the government of Ghana provided free health care at the public health centres, in an effort to make health care more accessible to majority of the population. This was mostly financed through tax revenues and external donor support.

By 1970, the deteriorating state of the economy could no longer support a tax-based health financing system, and during the 1985 structural adjustments, the ‘cash-and-carry’ system was introduced to reduce government expenditure in the health sector. Under this system, individuals were required to pay for their medical care at the point of service, which created a financial barrier to health care provision for the poorer citizens of the country.

In 2003, the National Health Insurance Scheme (NHIS) was launched to abolish the cash-and-carry system. Financed primarily by the National Health Insurance levy (2.5% of V.A.T.) and other secondary sources, the NHIS is aimed at reducing the out-of-pocket expenses at points of service. The free maternal health care was introduced in 2008, and integrated into the NHIS by 2010. This included free antenatal, postnatal and neonatal services. Since the inception of the NHIS, women who have health insurance are more likely to attend formal antenatal care check-ups (Brugiavini and Pace, 2011). The present study examines the importance of possessing health insurance in explaining variations in prenatal care visits, at the village-level. Other socioeconomic characteristics are taken into account, in addition to the effect of social forces.

### Social influence and antenatal care use

Features of local areas may influence health and collective social functioning and practices [14]. Shared norms and mutual trust among members of a society may be expected to promote communal action. Indeed, members of common social networks may share opinions and experiences and then act according to suggestions and general expectations. Therefore, a woman’s social network has the potential to play a significant role in her decision to use prenatal care services.

Most of the empirical studies on antenatal care and other health utilization services have emphasized the importance of individual characteristics, without paying as much attention to the societal impact [14,15]). Using data on a sub-sample of pregnant women in rural California, Chandler [16] finds that lack of social support predicted late entry of prenatal visits. Heaman et al. [14] use district-level data on 485 districts in Manitoba and find evidence of neighbourhood-level factors on the utilization of health care.

Using data on 34 neighbourhoods in Brazil, Lamarca et al. [17] find that women between their first trimesters of pregnancy and postpartum who have strong social support and are part of social networks reported that they had good health during this period, emphasizing the importance of an individual’s social network in influencing health behaviours and outcomes. Leal et al. [18] also investigated the importance of social support (including information support, religious activities, relatives and friends, neighbourhood association) among pregnant women in Brazil and found that these were significant social determinants for the appropriate use of prenatal care.

Although research conducted on Ghana’s NHIS has examined its impact on antenatal care visits, no research of which we are aware, has examined the impact of societal influences in women’s predisposition to attend antenatal care visits. According to Kieffer et al. [19] “area-level analyses contribute to program planning by identifying relevant social and environmental characteristics that may influence individual decisions to seek health care.” The present study uses 2008 GDHS data at the village-level to examine the impact of social influence on the frequency of antenatal care visits, controlling for a host of socioeconomic factors.

## Methods

The study uses data from the 2008 Ghana Demographic and Health Survey, which is a nationally representative dataset with data collected from all ten regions of the country. It uses information on women who have had children born in the last five years prior to the interview. In addition to socioeconomic and geographical information, there is also GIS information available for all surveyed villages. Table 1 summarises the study variables employed in the research. Although information is available at the individual level, variables have been aggregated to the village level in order to perform village-level spatial analyses. The independent variables included are based on prior empirical studies, particularly Arthur [5].

**Table 1:** Descriptive statistics of study variables

**Variables**	**Mean**	**Standard deviation**	**Minimum**	**Maximum**
**Dependent variable**				
No. of antenatal visits	6.056	2.057	1	13.25
**Health insurance?**				
Yes	0.429	0.324	0	1
No	0.571	0.324	0	1
**Number of children**				
No. of living children	3.134	1.020	0.5	7.25
**Distance as barrier?**				
Yes	0.298	0.319	0	1
No	0.702	0.319	0	1
**Transportation as barrier?**				
Yes	0.296	0.328	0	1
No	0.704	0.328	0	1
**Age**				
Age of woman	30.20	3.360	21.667	43
**Educational level of mother**				
at least primary school education	0.706	0.324	0	1
No education	0.294	0.324	0	1
**Wealth Quintiles**				
Poorest	0.224	0.350	0	1
Poorer	0.194	0.251	0	1
Middle	0.188	0.246	0	1
Richer	0.221	0.274	0	1
Richest	0.173	0.291	0	1
**Residence**				
Urban	0.431	0.496	0	1
rural	0.569	0.496	0	1
**Employment status**				
Employed	0.797	0.291	0	1
Not employed	0.203	0.291	0	1
**Region**				
Western	0.094	0.292	0	1
Central	0.086	0.281	0	1
Greater Accra	0.137	0.344	0	1
Eastern	0.107	0.309	0	1
Northern	0.091	0.289	0	1
Ashanti	0.165	0.372	0	1
Brong Ahafo	0.091	0.289	0	1
Upper East	0.071	0.257	0	1
Upper West	0.076	0.266	0	1
Number of observations	394 villages			

The unit of observation is the village and 394 villages are employed in the spatial analysis. The dependent variable is the average number of antenatal visits by women in each village and on average, women make six visits to the antenatal clinic during their pregnancy. On average, about 43% of women in these villages have health insurance and are eligible for free maternal health care. Previous studies have found a positive relationship between possession of health insurance and the frequency of antenatal care [5]. The number of living children that a woman has is included as a proxy for a woman’s familiarity with pregnancy and antenatal care. Women who have had a large number of children may be familiar with the pregnancy process and therefore may consider antenatal care less necessary [9]. Women who have had a lot of children may also attend fewer antenatal care visits if they have had a negative experience with earlier pregnancies. Women with more children may also underutilize maternal health care services as a result of many demands on their time, in addition to potential resource constraints [20]. Women in the study have an average of about 3 children, and a maximum of about 7.

Information is provided on the proportion of women who report that distance and transportation are barriers to their use of medical facilities. Almost 30% of the women in the sample report that both distance and transportation to medical care facilities is a problem. This is expected to have a negative impact on the intensity of antenatal visits [9]. The information on distance and transportation as barriers to medical services utilization are used as proxies for supply-side factors which may influence the intensity of women’s antenatal care utilization. It is reasonable to deduce that women who report transportation as a barrier to medical services utilization may not have ready access to health care facilities.

The average woman in the study is 30 years of age, with ages ranging from between 22 and 43 years of age. Age may have a positive impact on the frequency of antenatal visits if women are concerned about the possibility of birth complications with increased age. On the other hand, older women who have had many children may reduce the frequency of antenatal visits since they may be more experienced with pregnancy, and find antenatal visits unnecessary.

Information is also included on the educational status of women. In the study, over 70% of women have had at least primary school education. The level of education is a key factor that may be expected to affect a woman’s utilization of maternal health services [5,9] note that women’s attitude to antenatal care is influenced by their schooling- educated women are expected to be more efficient in their use of health care services. They are more aware of and open to using more modern methods of treatment. In addition, educated persons may adopt a more health-conscious approach to ensure continued good health [5].

Information is also provided on the wealth status of women; wealth is categorized by 5 variables- poorest, poorer, middle, richer and richest. 22% of the sample falls into the ‘poorest’ category while 19.4%, 18.8%, 22.1% and 17.3% fall into the other respective categories. Other researchers have found that wealth is a significant determinant of antenatal care utilization. Richer women appear to use these services more often than poorer women, even when access to health insurance is controlled for [21,5].

Urban dwellers are typically expected to use antenatal care services more frequently than women who live in rural areas, due to greater proximity to healthcare facilities in urban areas [10]. About 43% of the women surveyed live in urban areas. Working women who contribute to the household wealth may be expected to enjoy higher statuses within their homes and may be more likely to take individual decisions regarding health care use. In addition, working women are more empowered and able to pay for their health care. In addition, they are more likely to take advantage of modern methods of treatment in the event of a pregnancy complication [22]. Therefore, employed women may visit antenatal care clinics more intensively than unemployed women. From Table 1, almost 80% of the total sample is engaged in some form of occupation.

Dummies are also created for the regions in which women live in order to control for regional variations in the use of antenatal care. Regions that have more access to health care facilities may be expected to have a higher intensity of antenatal visits.

### Empirical methodology

A simple OLS regression is employed as a base model. The average number of antenatal visits for each village is regressed on a set of control variables, including a dummy variable for women who have health insurance. This specification is given as:

$$y_{i}=X_{i}\beta +u$$

Where *y*_*i*_ is the dependent variable and represents the average number of antenatal visits by women who live in a given village i, *X*_*i*_ the set of socioeconomic factors in the given village i that influence the dependent variable and *u* is the error term.

Spatial autocorrelation is present when women with high (low) levels of antenatal care visits are surrounded by other women with similarly high (low) antenatal care visits. The spatial lag model is used to correctly specify a model in which the dependent variable is found to be spatially autocorrelated. The spatial lag model specification is given by:

$$y_{i}=\mathrm{\rho W}y_{j}+X_{i}\beta +u$$

where *y*_*i*_ is the dependent variable (average number of antenatal visits by village i), *y*_*j*_ is the average number of antenatal care visits by neighbours of village i. *X*_*i*_ the set of socioeconomic factors in village i that influence the dependent variable. *ρ* is the spatial lag coefficient and *W* is the spatial weights matrix (specifies who each woman’s neighbours are). A distance band is created around each village such that each village has at least one neighbour. Within this distance band, villages that are farther apart from each other are constrained to matter less than villages that are closer to each other. *u* is the error term. The conceptual basis for this inverse distance weighting system is based on Tobler’s [23] first law of geography, which states that “Everything is related to everything else, but near things are more related than distant things.”

**Figure 1:**
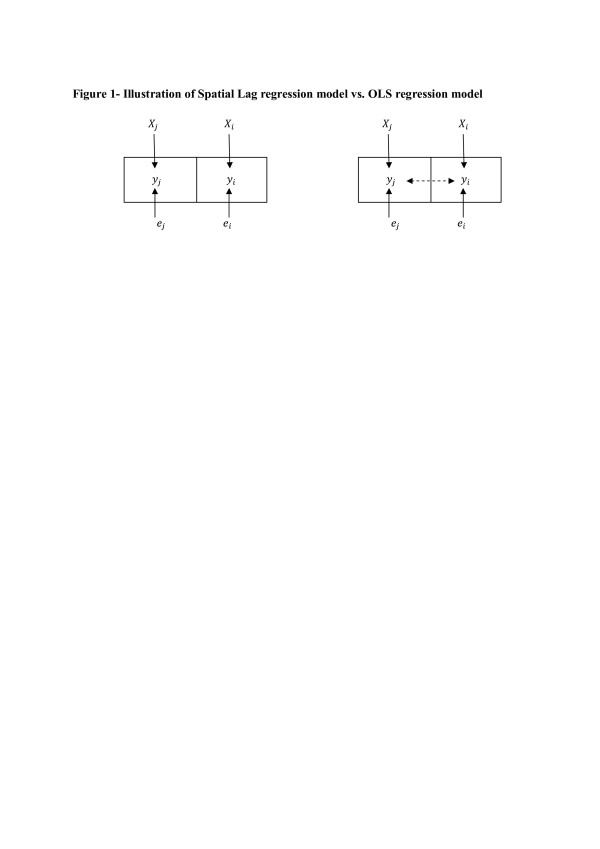
**Illustration of Spatial Lag regression model vs. OLS regression model.** Figure 1, adapted from Baller et al [24], provides some intuition of the difference between the spatial lag and simple OLS models. In the first diagram, each observation is influenced by its own set of structural/socio-economic factors; this is the OLS regression model. However, in the second diagram, each village’s maternal care utilization rate is allowed to be influenced by her neighbour’s maternal care utilization.

The spatial lag model specification allows the dependent variable (average number of antenatal visits for each village) to be influenced by the average number of antenatal visits of each village’s neighbours. Figure 1, adapted from Baller et al [24], provides some intuition of the difference between the spatial lag and simple OLS models.

In the first diagram, each observation is influenced by its own set of structural/socio-economic factors; this is the OLS regression model. However, in the second diagram, each village’s maternal care utilization rate is allowed to be influenced by her neighbour’s maternal care utilization. This relationship is captured by the spatial lag co-efficient, (*ρ*). A statistically significant spatial lag coefficient (*ρ*) would suggest that even when all of the usual explanatory variables are included in the regression model, spatial autocorrelation is still present and therefore, women’s use of maternal services in one village is correlated with the use of maternal services of other women in neighbouring villages.

### Exploratory spatial data analysis

**Figure 2:**
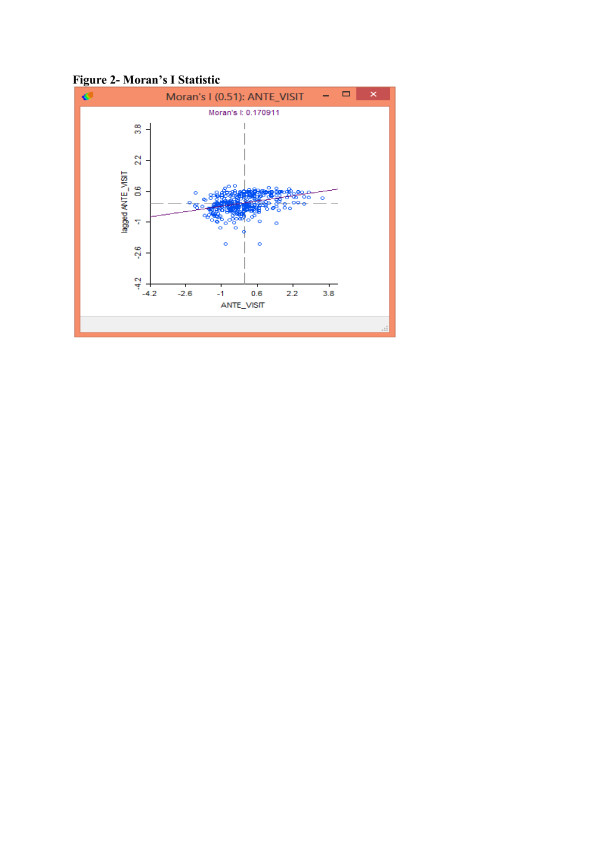
**Moran’s I Statistic.** The exploratory spatial data analysis is begun with the construction of a Moran’s I scatter plot, shown in Figure 2. This has the variable of interest (number of antenatal care visits) on the x-axis and the spatial lag (weighted average of neighbouring villages’ antenatal care visits) on the vertical axis. The Moran’s I statistic is a ‘global’ measure of spatial autocorrelation over the entire sample population. A positive Moran’s I statistic indicates a positive spatial autocorrelation where villages with a high (low) number of antenatal visits (given as ante_visit in Figure 1) are surrounded by other villages with similarly high (low) number of antenatal visits. A negative Moran’s I statistic indicates a negative spatial autocorrelation where villages with a high (low) number of antenatal visits are surrounded by other villages with low (high) number of antenatal visits. The positive Moran’s I statistic of 0.170911 shown in Figure 2 indicates that there is positive spatial autocorrelation, implying that women’s behaviours in each village may be influenced by other women’s behaviours in surrounding villages.

The exploratory spatial data analysis is begun with the construction of a Moran’s I scatter plot, shown in Figure 2. This has the variable of interest (average number of antenatal care visits for a given village) on the x-axis and the spatial lag (weighted average of neighbouring villages’ antenatal care visits) on the vertical axis. A regression line is constructed from the regression of the spatial lag on each given village’s intensity of antenatal care utilization, from which a positive or negative Moran’s I statistic is generated. The Moran’s I statistic is a ‘global’ measure of spatial autocorrelation over the entire sample population, and a positive Moran’s I statistic indicates a positive spatial autocorrelation where villages with a high (low) number of antenatal visits (given as ante_visit in Figure 1) are surrounded by other villages with similarly high (low) number of antenatal visits. A negative Moran’s I statistic indicates a negative spatial autocorrelation where villages with a high (low) number of antenatal visits are surrounded by other villages with low (high) number of antenatal visits.

The positive Moran’s I statistic of 0.170911 shown in Figure 2 indicates that there is positive spatial autocorrelation, implying that women’s behaviours in each village may be influenced by other women’s behaviours in surrounding villages.

**Figure 3:**
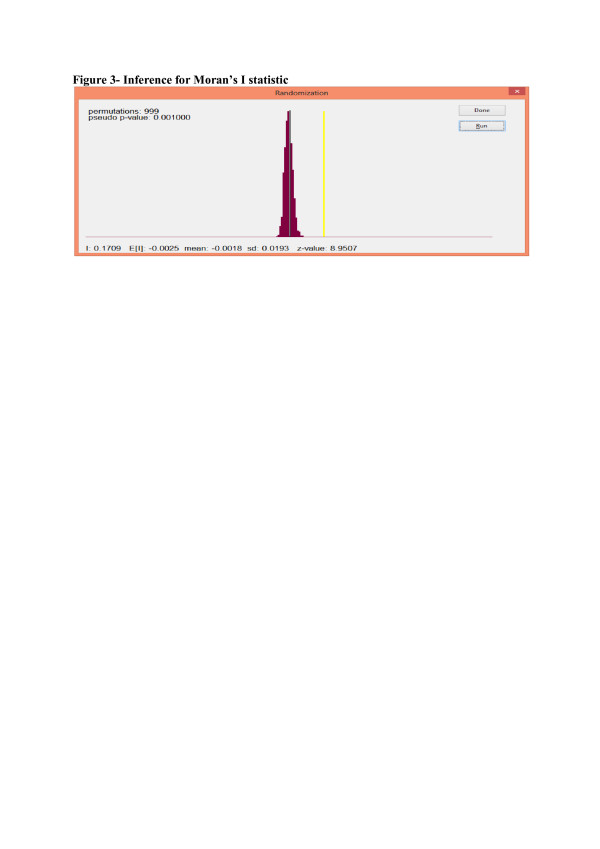
**Inference for Moran’s I statistic.** Inference for the Moran’s I is based on a random permutation procedure which recalculates the Moran’s I statistic a number of times and then generates a reference distribution. This is illustrated in Figure 3. The initial Moran’s I (yellow vertical line in Figure 3) is then compared to this reference distribution (brown distribution in Figure 3), and a *pseudo significance* level is calculated [25]. The Moran’s I statistic is highly significant at the 0.1% significance level, after 999 permutations. This implies that the observed positive spatial autocorrelation is very significant.

**Figure 4:**
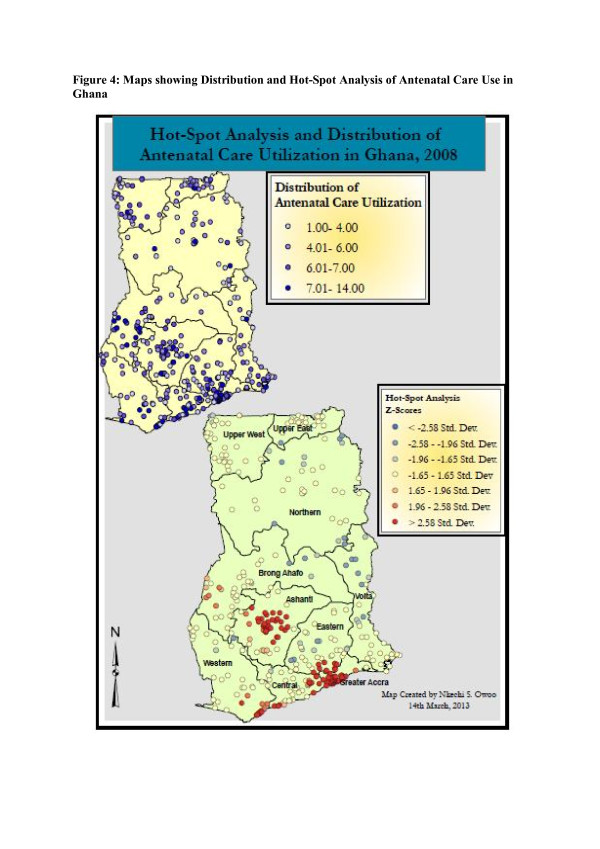
**Maps showing Distribution and Hot-Spot Analysis of Antenatal Care Use in Ghana.** The first map in Figure 4 illustrates the distribution of antenatal care in Ghana at the various surveyed villages, in addition to a hot-spot analysis, illustrating spatial autocorrelation on a more localized scale. Villages with the highest averages of antenatal care utilization are clustered in parts of the Ashanti, Upper East and the Greater Accra region. The northern region does not appear to have a high degree of antenatal care visits. The hot-spot analysis presents a map of the study area that is colour coded by the type of spatial autocorrelation and significance of the observed relationship. Dark-red clusters are villages of high antenatal visits, surrounded by other villages with similarly high antenatal visits. These clusters are found in the Ashanti region and parts of the Central, Greater Accra Brong Ahafo and Western regions. The darker shade of red implies a more significant spatial relationship. Blue clusters signify villages with low antenatal care utilization surrounded by other villages with similarly low antenatal care utilization. These are observed in the northern portions of the Volta region, with darker shades of blue signifying higher significance levels.

Inference for the Moran’s I is based on a random permutation procedure which recalculates the Moran’s I statistic a number of times and then generates a reference distribution. This is illustrated in Figure 3. The initial Moran’s I (yellow vertical line in Figure 3) is then compared to this reference distribution (brown distribution in Figure 3), and a *pseudo significance* level is calculated [25]. The Moran’s I statistic is highly significant at the 0.1% significance level, after 999 permutations. This implies that the observed positive spatial autocorrelation is very significant. Moran’s I may also be calculated at the local level in order to establish which clusters of villages are driving the observed spatial autocorrelation. This is shown in Figure 4, using a hot-spot analysis.

The first map in Figure 4 illustrates the distribution of antenatal care in Ghana at the various surveyed villages, in addition to a hot-spot analysis, illustrating spatial autocorrelation on a more localized scale. Villages with the highest averages of antenatal care utilization are clustered in parts of the Ashanti, Upper East and the Greater Accra region. The northern region does not appear to have a high degree of antenatal care visits.

The hot-spot analysis presents a map of the study area that is colour coded by the type of spatial autocorrelation and significance of the observed relationship. Dark-red clusters are villages of high antenatal visits, surrounded by other villages with similarly high antenatal visits. These clusters are found in the Ashanti region and parts of the Central, Greater Accra Brong Ahafo and Western regions. The darker shade of red implies a more significant spatial relationship. Blue clusters signify villages with low antenatal care utilization surrounded by other villages with similarly low antenatal care utilization. These are observed in the northern portions of the Volta region, with darker shades of blue signifying higher significance levels.

These spatial clusters indicate the possibility of social influence among neighbouring villages in terms of their antenatal care utilization. A finding of spatial correlation at the village level may be indicative of social interactions and influence among individual women that is captured on a more aggregated scale. For instance, in Ghana, most women communicate with each other when they attend social events like marriage ceremonies, funerals and festivals. During these events, women have the opportunity to exchange information relating to various aspects of their lives, including experiences with antenatal care and related pregnancy outcomes.

Villages may also be spatially correlated in terms of their antenatal care use if women from different villages have an opportunity to meet and interact often with each other, which increases the opportunity for, and probability of, the exchange of ideas, including opinions on maternal health care utilization. Such an opportunity in Ghana may be through each surrounding village’s market day. On each village’s market day, female traders and customers from neighbouring villages arrive to sell and purchase goods and services, respectively. This creates an enabling environment for the exchange of information, potentially relating to health practices. Additionally, health care workers often take advantage of the large number of women present on these days to arrange their clinics- and often times, durbars- on these market days and educate women about the importance of antenatal care, family planning, child welfare, etc. This could be a potential reason for the observed spatial relationship among survey villages in the above exploratory spatial data analysis.

## Results

Regressions of the spatial lag model are presented in Table 2. The first model specification presents estimates from a simple OLS regression model, while the second includes the spatial lag variable. Controlling for a host of socioeconomic variables, the study examines whether social influences continue to play a role in determining the frequency of antenatal visits among villages.

**Table 2:** Regression estimates of OLS and spatial lag model specifications

**Variables**	**Model 1**	**Model 2**
	**OLS**	**Spatial lag**
**Health insurance**	0.563*	0.701**
% who have health insurance	(1.75)	(2.26)
**Number of children**	−0.158	−0.171
No. of living children	(-1.24)	(-1.40)
**Distance as barrier**	0.178	0.248
% who find distance to health facility a problem	(0.28)	(0.41)
**Transportation as barrier**	−0.804	−0.910
% who find transportation a problem	(-1.30)	(-1.53)
**Age**	0.077**	0.075**
Age of woman	(2.22)	(2.25)
**Educational level of mother (no education as base)**	0.318	0.310
at least primary school education	(0.80)	(0.82)
**Wealth Quintiles (poorest as base)**		
Poorer	0.522	0.523
	(1.09)	(1.14)
Middle	0.373	0.305
	(0.78)	(0.67)
Richer	1.651***	1.462***
	(3.25)	(2.99)
Richest	2.367***	1.977***
	(3.85)	(3.31)
**Residence (rural as base)**	0.267	0.208
Urban	(0.99)	(0.81)
**Employment status (unemployed as base)**	−0.391	−0.297
Employed	(-0.98)	(-0.78)
**Region (Northern as base)**		
Western	0.556	0.248
	(1.53)	(0.69)
Central	0.465	0.281
	(1.19)	(0.74)
Greater Accra	0.677*	0.008
	(1.72)	(0.02)
Eastern	−0.187	−0.376
	(-0.53)	(-1.11)
Ashanti	0.461	0.086
	(1.39)	(0.26)
Brong Ahafo	0.639*	0.509
	(1.80)	(1.50)
Upper East	0.822**	0.811**
	(2.02)	(2.09)
Upper West	0.723*	0.738**
	(1.93)	(2.07)
*p* (spatial lag co-efficient)	-	0.628***
		(3.59)
Number of observations	394	394
R-squared	38.02%	40.2%

Both model specifications show a positive and significant relationship between health insurance and the age of women, and the intensity of antenatal care visits. Women who have health insurance appear to have a greater intensity of antenatal care utilization. Older women also appear to have a larger number of antenatal care visits. These effects are stronger and more significant under the spatial lag than under the OLS regression model. Women belonging to the ‘richer’ and ‘richest’ wealth categories appear to utilize maternal care services more frequently than women in the ‘poorest’ category, controlling for possession of health insurance. This may imply that there may be other additional costs associated with maternal health care which the existing health insurance scheme may not adequately cater for [5].

Under the OLS model specification, women in the Greater Accra, Brong Ahafo, Upper East and Upper West regions appear to attend antenatal care more frequently than women in the northern region. Under the spatial lag model, women in the Upper East and Upper West regions appear to utilize antenatal care services more frequently than women in the Northern region. From the graphical depiction in Figure 1, it may be observed that the utilization of antenatal care services in the Northern region is low. This may be as a result of a lower level of formal education of women in the Northern region (22%), compared to women in the Upper East (40%) and Upper West (41%), which may influence the use of antenatal care services among these regions. Regional effects are smaller under the spatial lag regression model once underlying spatial effects are controlled for. This implies that the regional differences in antenatal care utilization are over-estimated when spatial effects are not controlled for using a simple OLS regression model.

The spatial lag co-efficient in model 2 is positive and highly significant, controlling for women’s geographical and socioeconomic factors. A positive spatial lag co-efficient implies spatial autocorrelation between a given village and its surrounding neighbours. Villages with high (low) levels of antenatal care use are surrounded by other villages with similarly high (low) levels of antenatal care use. This might imply a degree of social influence operating among these villages and contributing to the observed similarities in their intensity of antenatal care utilization.

The presence of spatial correlation among villages in Ghana makes the spatial lag model a better model for the analysis of antenatal care utilization by women, as evidenced by the higher explanatory power of the spatial lag model. Models that attempt to study the factors that influence maternal care use (in Ghana) without controlling for spatial effects are potentially mis-specified.

## Discussion

### Socioeconomic factors

The results showed a positive relationship between a woman’s age and her intensity of antenatal care use. This is consistent with other research finding [6,22,26,27]. A reason for this may be that older women are more aware of the benefits of antenatal care and are therefore more likely to use these services more intensively.

Women who have access to health insurance also have a greater intensity of antenatal care utilization. This finding is also reported in Arthur [5] and Brugiavini and Pace [13]. Health insurance is expected to mitigate out-of-pocket expenses related to maternal health care utilization, which will encourage more women to utilize these services more readily.

The results of the regression also suggested that compared to women in the poorest wealth category, women in the richer and richest wealth categories utilize maternal health services more intensively. Similar to findings by Arthur [5], wealth plays a significant role in antenatal care utilization, even controlling for health insurance. This may imply that there are other costs related to antenatal care visits that are currently not catered for under the health insurance scheme. The findings of a positive effect of wealth on antenatal care utilization are supported by Gage [28], Fosu [21], Chakraborty et al. [22] and Arthur [5].

Given differences in access to health care services and infrastructure in Ghana, the region in which a women lives is expected to have an impact on the intensity of her use of antenatal care services. Compared to women in the Northern region, women in the Greater Accra, Brong Ahafo, Upper East and Upper West regions appear to use antenatal care services more intensively. Only the upper east and upper west regions remain significant under the spatial lag model specification, when village-level spatial effects are controlled for. Government may take steps to improve upon the distribution of health facilities in the various regions in Ghana in order to encourage greater utilization of maternal health services in the country.

### Social influences

This study was aimed at determining the effect, if any, of social forces in women’s decision to utilize antenatal care services in Ghana, controlling for a host of socioeconomic and geographic factors. The results indicate that women’s utilization of antenatal care services is spatially correlated among villages in Ghana, implying a degree of interaction and possible influence among these women.

In Ghana, most women communicate with each other when they attend social events like marriage ceremonies, funerals and festivals. They may exchange information with each other on the benefits of prenatal care and the resultant positive pregnancy outcomes, which may encourage other women to also patronize these maternal care services. According to Fukuyama [29], the benefits that individuals enjoy through interactions with their social groups can result in a decrease in inadequate prenatal care on a broader scale such as at the village level.

Another opportunity for women to interact with one another occurs on the various market days for each of the surrounding villages. This is a potentially effective channel of inter-village communication given that in one week, a woman may have visited about three of the surrounding villages. This may be expected to increase the scope and rate of information dissemination. Additionally, health care workers often take the opportunity to schedule their clinic sessions- and often times, durbars- on these market days to educate women about the importance of antenatal care.

Social influence may also occur from the activities of certain ‘leaders’ in the community such as Queen mothers. If Queen mothers understand and applaud the use of antenatal care in their respective villages, other followers in her village and other neighbouring villages might copy this behaviour. This finding is important for health policy in Ghana. Dissemination of relevant information may be done using women’s social networking channels such as market place gatherings, churches, and other social groups, where women regularly meet and exchange information and experiences. In addition, female ‘leaders’ such as Queen mothers could be involved in medical outreach programs and encouraged to emphasize the importance of antenatal care to women in their communities in an effort to reducing the rate of maternal mortalities in these villages and in Ghana, at large.

Finally, social influence may be present among villages in Ghana that have similar religious, cultural and traditional values. Here, women in these villages may behave similarly as a result of shared beliefs, norms and practices that influence their behaviours. It is important to note however, that where villages behave similarly despite limited interactions among them, social influence cannot be said to have taken place. Social influence is conditional on interactions among individuals which encourages similar choices and behaviours.

The study has a few other limitations that must be considered in the interpretation of the results. First, it is important to note that the observed spatial autocorrelation may be as a result of underlying cultural and/or socio-economic factors which may be influencing the observed spatial patterns. As emphasized by Pickett and Pearl [30], “without adequate control of individual socioeconomic status, neighbourhood level effects may act as proxies for unmeasured aspects of individual SES”. For instance, in high-education regions like Greater-Accra and Ashanti regions, women may use antenatal care more intensively. Although this may be observed as a spatial cluster of high antenatal care utilization, education would be the driving force behind the intensity of antenatal care utilization, and not a process of information exchange and influence as assumed. Similar arguments can be made for culture and religious factors as underlying driving forces. Although a large number of socioeconomic and geographic controls are included in the regression, these controls are inexhaustive and the observed spatial autocorrelation may pick up certain neighbourhood effects such as general access to medical facilities. Second, researchers who engage in spatial analyses as this are encouraged to provide some basis for their choice of neighbourhood constructs. This paper uses an inverse distance weighting system given that locations that are closer to one another are more likely to influence each other through networks and connections reinforced by shared cultural factors such as language, ethnicity and religion [23]. Thirdly, this paper does not explicitly control for supply-side variables such as the doctor-patient ratio and the distance to health facilities as a result of data limitations. However, the variable on whether or not distance or transportation is a problem to women’s access to health care facilities may be used as a proxy for the distance to health facilities.

## Conclusion

The study investigated the determinants of maternal care utilization in Ghana using data from the 2008 Ghana Demographic and Health Survey. In addition to examining the effects of health insurance, wealth, education and geographical variables on antenatal care use, the research also controlled for the presence of spatial effects among villages. In spite of the strong correlation between health insurance and other factors such as wealth, woman’s age, and antenatal care utilization, an independent connection was observed between social influences and antenatal care utilization at the village level.

From a policy perspective, programs which are aimed at increasing women’s participation in antenatal care utilization may take advantage of certain social networks in disseminating relevant information about medical care. Mass media such as TV and radio may also be used since women may discuss the new information and their own experiences with each other at regular social events. In addition, ‘leaders’ in the various villages such as Queen mothers may also be included in medical outreach programs in an attempt to encourage better use of medical services in general, and antenatal services in particular.

## Competing interests

The authors declare that they have no competing interests.

## Authors’ contributions

Nkechi Srodah Owoo conceived of the study. Both Nkechi Srodah Owoo and Monica Puoma Lambon-Quayefio undertook the analysis- a greater portion of the spatial analysis was undertaken by Nkechi Srodah Owoo. Nkechi Srodah Owoo did the write-up of the analysis. Both authors read and approved the final manuscript.
